# Efficient Production and Experimental Analysis of Bio-Based PLA-CA Composite Membranes via Electrospinning for Enhanced Mechanical Performance and Thermal Stability

**DOI:** 10.3390/polym17081118

**Published:** 2025-04-20

**Authors:** Irfan Farooq, Abdulhamid Al-Abduljabbar

**Affiliations:** Department of Mechanical Engineering, King Saud University, Riyadh 11421, Saudi Arabia; 443107001@student.ksu.edu.sa

**Keywords:** electrospun biopolymer composite, biodegradable membranes, PLA-CA composite, mechanical properties, thermal stability, nanofibrous membranes, hydrophobic

## Abstract

Environmentally friendly biopolymer nanofibrous composite membranes with enhanced mechanical properties and thermal stability were fabricated via electrospinning with different compositions of polylactic acid (PLA) and cellulose acetate (CA). Firstly, PLA and CA composite membranes were prepared and optimized. Then, the optimized membranes were annealed at temperatures ranging from 80 °C to 140 °C, for annealing times between 30 and 90 min. The developed membranes were characterized by FE-SEM, XRD, FR-IT, TGA, DSC, tensile testing, water contact angle, and resistance to hydrostatic pressure. PLA 95-CA 5 was the optimum composite, with a tensile strength 9.3 MPa, an average fiber diameter of 432 nm, a water contact angle of 135.7°, and resistance to a hydrostatic pressure of 16.5 KPa. Annealing resulted in further improvements in different properties. The annealed membranes had thermally stable microporous structures, without shrinkage or deterioration in nanofiber structure, even at an annealing time of 90 min and an annealing temperature of 140 °C. By increasing either the annealing time or temperature, the crystallinity and rigidity of the nanofiber composite membranes were increased. The annealed membrane demonstrated a tensile strength of 12.3 MPa, a water contact angle of 139.2°, and resistance to a hydrostatic pressure of 36 KPa. Electrospinning of PLA-CA composite membranes with enhanced mechanical properties and thermal stability will pave the way for employing PLA-based membranes in various applications.

## 1. Introduction

Demand for disposable plastics is continuously increasing day by day. Technological advancement in the petrochemical-based industry has tried its best to meet the global demand. However, the non-degradable characteristics of petroleum-based polymers have drastic effects on the global ecosystem. The environmental impacts of plastic product waste have increased worldwide concerns, due to the limited disposable techniques that can be utilized. The incineration of these plastic wastes results in the production of large amounts of carbon dioxide, toxic chemicals, and gases, which constitute a major factor influencing the increase in global warming and global pollution. Petroleum-based resources and satisfactory sites for waste landfills are also limited. On the other hand, the production of polymers from renewable resources is preferred from the perspective of life cycle assessment [1,2]. The utilization of biopolymers can appreciably reduce greenhouse gas emissions and the consumption of fossil energy, in comparison to traditional petrochemical polymers. The development of environmentally friendly and degradable plastic materials has attracted a lot of attention in academia and on an industrial scale.

The research community in the field of membrane technology has become more conscious about the detrimental impacts of petroleum-based polymers on public health and the environment. European Bioplastics and the Nova-Institute published general recommendations in 2019 to encourage the utilization of sustainable products by boosting the usage of bio-based materials. This awareness resulted in much interest in the progressive development of biopolymers to replace petroleum-based polymers. Recently, biopolymers such as poly (lactic acid) (PLA), poly (hydroxyalkanoates) (PHA), polyglycolic acid, polybutylene succinate, poly ethyl lactone, chitosan, cellulose, chitin, and poly (ethylene glycol) (PEG) have attracted much attention, due to their ecological, biocompatibility, and degradability properties, and as a result, are considered much superior alternatives to petroleum polymers [3,4,5].

PLA is the most promising, sustainable, and biodegradable material that has the ability to replace non-degradable petroleum-based materials. PLA is a linear aliphatic thermoplastic polyester, commonly generated by a process of ring-opening polymerization of lactic acid, which is produced by fermentation of corn starch, sugar beet, sugar cane, rice, etc. It is available abundantly, and has good characteristics, such as biocompatibility, biodegradability, excellent spin ability, low carbon footprint, low cost, sunlight resistance, and antimicrobial activity [4,6,7]. Recently, PLA has been examined for numerous applications, such as filtration masks, water treatment, tissue engineering, drug-controlled release, scaffolds, implants, food packaging, and agricultural products. However, PLA has less thermal stability, low tensile strength, brittleness, and lower elongation at break, which hinders its utilization in various applications [3,4]. Cellulose acetate (CA) is a biopolymer that has tunable mechanical characteristics, and is used to enhance the tensile strength and toughness of composite materials, reducing their vulnerability to brittle failure, and improving their thermal stability, biodegradability, and sustainability [8,9,10]. Therefore, CA is a good candidate for the enhancement of the mechanical and thermal properties of composite materials.

There are various techniques that can be employed to process PLA-based materials, such as solvent casting [11], melt spinning [12], dry spinning [13], electrospinning [3,14,15,16], etc. Among these techniques, electrospinning is considered a facile process to generate micro/nanofibers, and it has gained much attention in academia and on an industrial scale, due to its simplicity in processing. The micro/nanofibers can be spun into nonwoven structures that have a high surface-area-to-volume ratio, high efficiency, and are able to fabricate the desirable porous microstructure, etc. [17]. The characteristics of electrospun membranes can be easily manipulated by regulating polymer solution, environmental, and electrospinning process parameters [5]. Generally, the electrospinning process can produce fibers with diameters ranging from 50 nanometers to 1 micrometer, although some researchers have produced very thin nanofibers with diameters as low as 1 nm [18].

Electrospun PLA-based membranes have been utilized for numerous applications, such as drug delivery systems [19], scaffolds [20], desalination [21], protective textiles [15], micro/nanofiltration [22], etc. However, utilization of electrospun PLA membranes in commercial applications is limited, due to their low tensile strength, brittleness, low elongation at break, and lower thermal stability [22,23,24]. It is commonly recognized that the mechanical properties of polymers are highly affected by their crystalline structure. Generally, increasing crystallinity results in increased tensile strength and stiffness. However, quick solvent evaporation and polymer solidification during the electrospinning process restrict the crystallinity of fibrous structures. Furthermore, in a nonwoven fibrous structure, the loose connection between micro/nanofibers results in weak structural integrity of mats [25,26]. These factors are the major reason for the poor mechanical properties of electrospun PLA-based membranes, and constitute an obstacle to their utilization in different commercial applications. Therefore, there is a need to figure out an approach to enhance the mechanical properties and thermal stability of electrospun PLA membranes without compromising their inherent fibrous morphology.

Various techniques have been employed to enhance the mechanical properties of electrospun PLA nanofibrous mats, such as plasticization [25], chemical crosslinking [27], copolymerization [28], solution blending [7], annealing treatment [22,23], and hot pressing [29]. In an attempt to enhance PLA’s properties, it has been blended with different petroleum and biopolymers, such as cellulose acetate, cellulose nanofibrils (CNFs) [30], poly (ethylene glycol) [31], poly (vinyl pyrrolidine) (PVP) [32], polyvinylidene fluoride (PVDF) [33], etc. Gomaa SF et al. developed PLA/CA electrospun composite scaffolds, loaded with thymoquinone (TQ) as an antimicrobial agent, for wound dressing, by utilizing different concentrations of PLA and CA. They analyzed the effects of the fibrous microstructure, strength, hydrophilicity, bioactivity on wound dressing characteristics of the membranes [34]. Chen J et al. fabricated electrospun poly (lactic acid)/regenerated cellulose scaffolds for tissue engineering. They analyzed the biological activity, biomineralization, hydrophilicity, mechanical strength, and porosity of the membranes [35]. Zhu M et al. fabricated cellulose acetate (CA)/poly-L-lactic acid (PLLA)/Halloysite nanotube fibrous composite membranes for gel polymer electrolytes for utilization in lithium ion batteries. They analyzed the effects of the microstructure, thermal stability, and crystallization on electrochemical performance [36]. Abdullah et al., by utilizing coaxial electrospinning, fabricated core–shell fibrous membranes, with cellulose acetate as the shell and poly lactic acid as the core, for bone tissue scaffolds. They improved the biocompatibility and mechanical strength of the scaffolds [37]. Biopolymers can be utilized for a range of different applications; their biocompatibility, biodegradability, fibrous microstructure, porosity, mechanical strength, wettability, and thermal stability are important factors to be taken into consideration.

An appropriate annealing process might be more environmentally friendly. In the annealing process, the material is heated above the glass transition temperature, but below the melting temperature; then, it is allowed to cool down in the furnace to an ambient temperature, to induce structural changes. This paves the way for altering the polymer’s molecular structure and enhancing the degree of crystallinity, inducing overlapping and fusion between the nano/microfibers, resulting in the enhancement of the fibers’ integrity and an improvement in the mechanical characteristics of nanofibrous mats [23]. The conventional annealing process of PLA mats leads to fiber relaxation and shrinkage of membranes [22]. Riberio et al. performed annealing of electrospun PLA membranes at 140 °C, and reported severe deterioration of the fiber microstructure, concluding that it is challenging to generate the stable crystalline phase in a nano/micro system with such a high sensibility to thermal defects [38]. Viswanath et al. observed a similar phenomenon where the microstructure of nanofibrous mats was destroyed due to the annealing process, but in the case of photothermal annealing in the presence of embedded gold nanoparticles, the nanostructured morphology was preserved [39]. Bye et al. developed a vapor annealing technique involving suspending an electrospun membrane above a pool of dichloromethane [40]. Naga et al. demonstrated the solvent-induced crystallization of amorphous PLA [41]. Lin et al. enhanced the mechanical properties of an electrospun PLA membrane by the annealing process; at an annealing temperature of 105 °C, shrinkage occurred in the membrane, and it turned into a sheet [22]. However, the annealing process has an impact on the fiber morphology, hydrophobicity, and mechanical strength of electrospun membranes [42]. In many academic and commercial applications, the successful implementation of biopolymers with a synthetic nanostructure is majorly dependent on their mechanical behavior and morphology. Therefore, it is critical to choose the optimum set of parameters to achieve the best compromise in terms of the morphology, thermal stability, hydrophobicity, and mechanical strength of electrospun membranes. As a result, achieving a compromise between the required properties, along with gaining an understanding of the underlying mechanisms accountable for enhanced characteristics, are the main research challenges in field of electrospun composite membranes. In this study, PLA and CA blend systems were utilized to fabricate environmentally friendly, biodegradable electrospun composite membranes. The mechanical strength, thermal stability, crystallinity, chemical structure, hydrophobicity, breathability, and resistance to hydrostatic pressure of the resulting composite membranes were investigated. This research was divided into two phases. In the first phase, composite membranes were developed and optimized with different concentrations of PLA and CA. In the second phase, an annealing operation was performed on the optimized membranes. Annealing was performed under a specific set of annealing parameters: the annealing time was varied from 30 min to 90 min, and the temperature was varied from 80 °C to 140 °C. The morphology of as-spun and annealed composite membranes was determined with the SEM technique. Their thermal characteristics were analyzed with TGA and DSC analysis, and the effect of heat treatment on the crystallinity of the annealed composite membranes was evaluated with XRD analysis. Further, the strength of the as-spun and annealed composites was determined with tensile stress–strain analysis. The hydrophobicity of the membranes was measured with analysis of the water contact angle and resistance to hydrostatic pressure, and chemical structural analysis was performed with FT-IR analysis. We fabricated PLA-based biocomposite electrospun composite membranes with enhanced mechanical properties, thermal stability, and a stable porous microstructure, without any deterioration of fibrous morphology, at an elevated annealing temperature of 140 °C and an annealing time of 90 min.

## 2. Experimental

### 2.1. Materials

The base material for the nanofibrous membrane was polylactic acid (PLA M*_w_* = 163,000, 1.25 g·mL^−1^, LX175^®^, Filabot, Barre, VT, USA), and the additive composite material was cellulose acetate (CA), purchased from Sigma Aldrich (St. Louis, MO, USA). The solvents N, N-dimethylformamide (DMF) (analytical-reagent grade, ≥99.8% purity), dichloromethane (DCM), acetic acid, and acetone (≥99.5%) were utilized to prepare the solution for electrospinning, and were secured from Sigma Aldrich. Deionized water was used to check the water contact angle of the PLA-CA composite nanofibrous membranes.

### 2.2. Solution and Memerane Preparation

**Figure 1:**
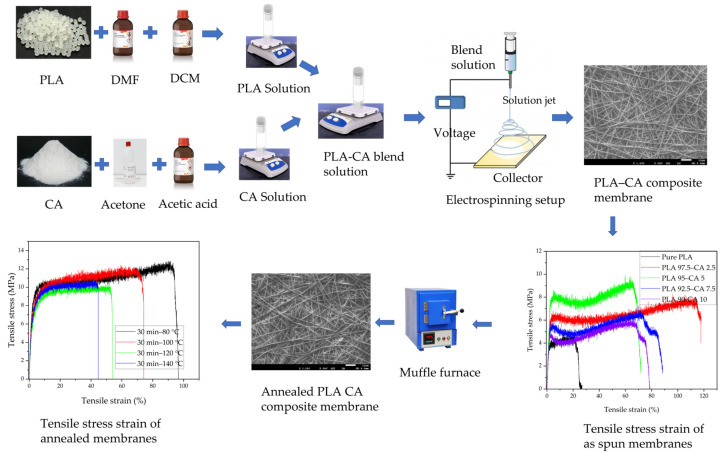
Process flow diagram for production of electrospun PLA-CA composite membranes.

The biopolymeric composite membranes were prepared by blending polylactic acid solution with cellulose acetate solution. Figure 1 presents a schematic flow diagram of the process of producing the composite membranes from the two solutions. Initially, the two separate PLA and CA solutions were prepared. The PLA solution had a 10 wt.% concentration of PLA, which was dissolved in a DCM and DMF mixture with a 4:1 volume ratio of solvents, respectively, kept at 40 °C and magnetically stirred for 3 h, and then cooled down to room temperature. As for the CA solution, it had a 17 wt.% concentration of CA, which was dissolved in an acetone and acetic acid mixture with a 3:1 volume ratio of solvents, respectively, kept at 45 °C, stirred for 6 h, and then cooled down to room temperature. After preparation of both solutions, the blend solution was prepared by mixing the CA solution into the PLA solution in varying concentrations, from 0 to 10%, and keeping this at room temperature on a magnetic stirrer for 24 h, to obtain a homogeneous solution. The solution was processed with an electrospinning machine (NF-500, MECC, Fukuoka, Japan); the biopolymer solution was loaded in a 10 mL syringe with a needle diameter of 0.6 mm. It was processed at a feed rate of 0.8 mL/h and under an applied voltage of 18 KV, and the tip-to-collector distance was kept at 150 mm [43].

Initially, CA solution was blended with PLA solution at different concentrations, varying from 0 to 80% (*v/v*) concentration. Based on the water contact angle, the concentration range for the hydrophobic composite membranes was selected. PLA-based hydrophobic electrospun composite membranes were prepared by blending CA with PLA solution varying from 0 to 10% (*v/v*) concentration. The best membrane was selected based on tensile tests, hydrophobicity, resistance to hydrostatic pressure, and morphological analysis. The optimized membrane was selected for further annealing processes. The set of annealing conditions was chosen based on DSC analysis for glass transition and melting temperatures and SEM microstructure images of the composite membranes. The annealing process was carried out at annealing times of 30, 60, and 90 min and annealing temperatures of 80, 100, 120, and 140 °C. 

### 2.3. Characterization and Measurements

The morphology of the as-spun and annealed electrospun nanofibrous membranes was analyzed with a field emission scanning electron microscope (FE-SEM: JSM-7600, JEOL, Tokyo, Japan). Small membrane specimens were cut off and affixed on the stub with carbon tape. To enhance the electrical conductivity of the membrane specimens, which is required during SEM analysis, they were coated with platinum. The coated specimens were placed in FE-SEM under a high vacuum, and their microstructures were analyzed at different magnifications. ImageJ 1.54j software was utilized to calculate the average pore size and average diameter of the fibers.

The tensile strength of the as-spun and annealed specimen membranes was analyzed by utilizing Instron apparatus (Electropuls, Bucks, UK). The gauge length of the membrane specimens was kept at 20 × 45 mm, and the test was run at a crosshead speed of 5 mm/min, according to ASTM Standard D882 [44]. To facilitate the handling of membrane samples during experimentation, all specimens were first adjusted on paper frames. Hydraulic grips were utilized to fix the samples in a machine load cell. After gripping, the paper frame was cut with the help of scissors, leaving only the tensile sample of membrane between the grips. The average values of Young’s modulus (E), tensile strength (σ), and elongation at break (ε_b_) were calculated from stress–strain curves.

The thermal properties of the as-spun and annealed biopolymer membranes were determined using thermal gravimetric analysis (TGA) and differential scanning calorimetry (DSC) equipment (Q600, TA Inc., New Castle, DE, USA). A 10 to 12 mg specimen was cut and placed in a ceramic pan in apparatus under ramp conditions, in a temperature range from 25 °C up to 600 °C, at a heating rate of 10 °C min^−1^. All specimens were processed in a nitrogen gas environment. A Muffle furnace was used for annealing treatment of the specimens. The specimens were placed between two glass slides and heated at a specific annealing temperature and time, before being cooled down in the furnace until room temperature was reached, i.e., 25 °C. The amorphous and crystalline natures of the as-spun and annealed composite membranes were analyzed with X-ray diffraction analysis (XRD-7000, Shimadzu, Kyoto, Japan). The 2θ degree range was applied from 5° to 90°, with a continuous scanning rate of 2°/min. The chemical structure of the pristine PLA, PLA-CA composite, and annealed membrane specimens was determined by Fourier transform infrared spectroscopy (FT-IR); the membranes were scanned in the range of 500 to 4000 cm^−1^.

The hydrophobic characteristics of the as-spun and annealed composite membranes specimens were analyzed by utilizing a CA goniometer (OCA 15EC, Data Physics, Hailsham, UK). The as-spun and annealed composite samples were subsequently tested for resistance to hydrostatic pressure and breathability. The hydrostatic pressure and breathability of the as-spun and annealed membranes were determined using a manual setup. For the breathability test, the composite membrane was adjusted in a filtration assembly flask, which was partially filled with water; compressed air was passed through the assembly flask, and the breathability of the membrane was analyzed by visualizing the air bubbles coming out of the water. The waterproofness of the composite membranes was determined by measuring the resistance to hydrostatic pressure; the manual setup was developed in lab as reported in [45]. The composite mat was fixed in a filtration assembly flask partially filled with tap water; hydrostatic suction pressure was generated with the help of a vacuum pump. The pressure on the membrane surface was changed by changing the suction pressure. The pressure was increased gradually until water droplets started falling from the bottom of membrane.

## 3. Results and Discussion

The results of the production technique of as-spun, annealed composite membranes and experimental analysis of different properties are presented. First, the scanning electron microscopy results are displayed to elaborate the morphology of the PLA-CA composite membranes. The average diameter of fibers, their normal distribution, and porosity were measured to examine the effects of different concentrations of blending polymers and of the annealing process. Thermal analysis was performed to calculate the thermal properties of the composite membranes with microporous microstructures at elevated annealing temperatures and times. The effect of annealing on the crystallinity of the composite membranes was verified with XRD analysis. The strength of the composite membranes was determined with tensile testing. The hydrophobic characteristics of the biopolymer membranes were examined by analyzing the water contact angle. Further, the strength and breathability of the membranes were verified through resistance to hydrostatic pressure and breathability experiments. Finally, the chemical structure of the PLA-CA composite membranes was studied with FT-IR analysis.

### 3.1. Morphology

**Figure 2:**
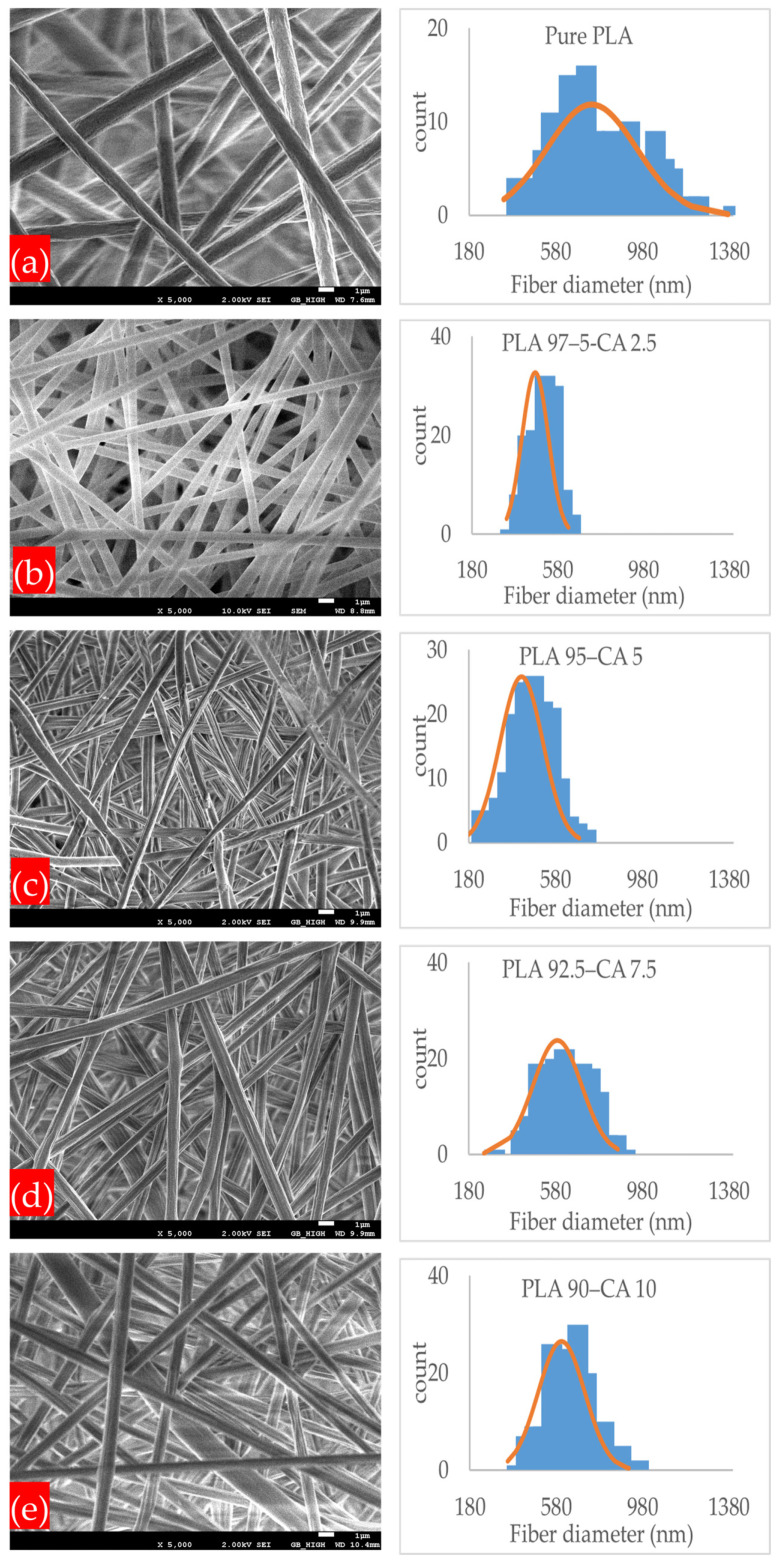
SEM microstructure images and normal distribution of fiber diameter at different concentration of CA in PLA-CA composite membranes. (**a**) Pure PLA; (**b**) PLA 97.5–CA 2.5; (**c**) PLA 95–CA 5; (**d**) PLA 92.5–CA 7.5; (**e**) PLA 90–CA 10.

The morphological aspects of pristine PLA and PLA-CA composite membranes were determined with SEM microstructural images, as shown in Figure 2a–e. All the membrane specimens had smooth, homogeneous, and bead-free nanofibrous microstructures. The average fiber diameter of the pristine PLA membrane was 746.3 nm, with a standard error of ±16.6 nm. Meanwhile, the average fiber diameter of all the PLA-CA composite membranes was lower than that of the pristine PLA. As the percentage of CA varied from 0 to 10%, the average diameter of the fiber kept changing. The average diameter for the PLA 97.5–CA 2.5 specimen was 475.7 ± 5.9 nm, and it decreased in the PLA 95–CA 5 specimen, to reach its lowest value of 432.1 ± 7.8 nm. Further increase of the concentration of CA in the PLA solution increased the average fiber diameter. The PLA 92.5–CA 7.5 and PLA 90–CA 10 specimens had an average fiber diameter of 588.2 ± 8.7 and 604.7 ± 8.1 nm, respectively.

**Figure 3:**
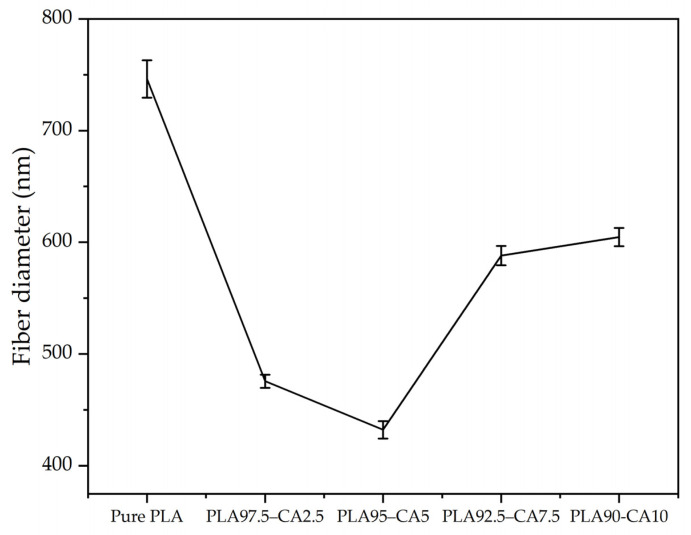
Average fiber diameter at different concentrations of PLA-CA composite membranes.

The average fiber diameter of the pure PLA and PLA-CA composite membranes is shown in Figure 3. The concentration of CA in the blend solution was the most influential factor in determining the average fiber diameter of the electrospun nanofibers.

From 0 to 5% CA concentrations, the PLA-CA composite solution had a lower viscosity and higher charge density, which led to thin fibers. However, as the CA concentration in the solution was further increased up to 10%, there was an increase in the blend solution’s viscosity, a reduction in charge density, and a slower evaporation rate of the solvent; accumulatively, all these factors resulted in thicker nanofibers. Therefore, the concentration of CA in the PLA-CA blend solution had a significant effect on the morphological, thermal, and mechanical properties [46,47].

The PLA 95–CA 5 composite membrane exhibited the lowest fiber diameter. Due to its nano characteristics, it had superior morphological, mechanical, and thermal properties, so it was selected as the optimized membrane. Annealing was performed on the PLA 95–CA 5 composite membrane. The set of annealing conditions was carefully selected to obtain the best combination of morphological, mechanical, thermal, and hydrophobic properties of electrospun mats based on differential scanning calorimetry (DSC) experimental results, which will be discussed later (in Figure 9c, and SEM microstructural images). Annealing was performed under different annealing times, such as 30, 60, and 90 min, and at different annealing temperatures, such as 80, 100, 120, and 140 °C.

**Figure 4:**
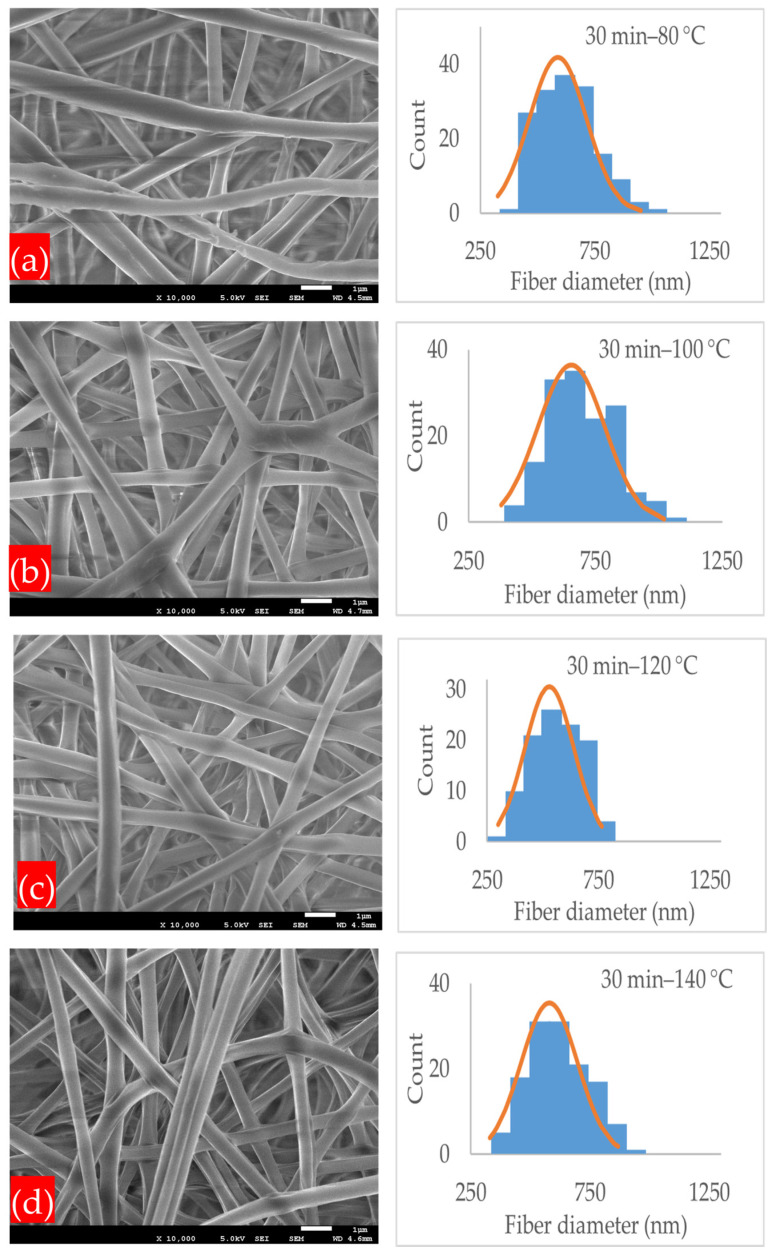
SEM microstructure images and normal distribution of fiber diameter of membranes annealed under following conditions: (**a**) 30 min–80 °C; (**b**) 30 min–100 °C; (**c**) 30 min–120 °C; (**d**) 30 min–140 °C.

**Figure 5:**
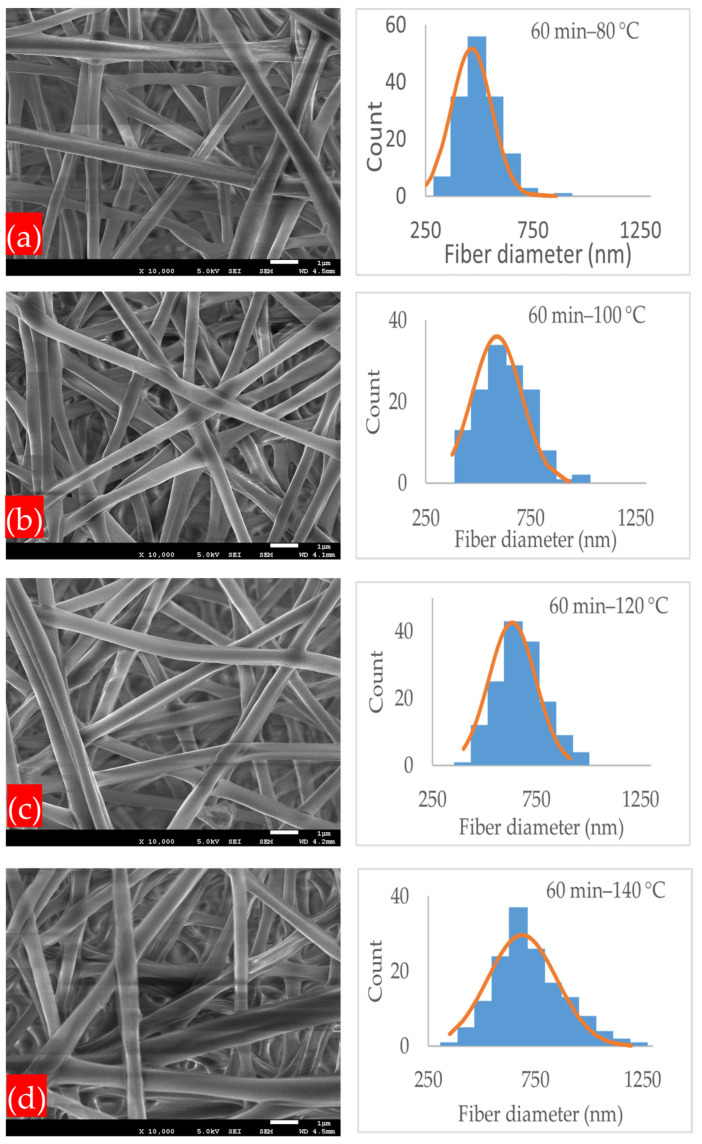
SEM microstructure images and normal distribution of fiber diameter of membranes annealed under following conditions: (**a**) 60 min–80 °C; (**b**) 60 min–100 °C; (**c**) 60 min–120 °C; (**d**) 60 min–140 °C.

Figure 4a–d demonstrates the microstructures and normal distribution of the fiber diameter of the electrospun nanofibrous membranes under the annealing time of 30 min and at different annealing temperatures of 80, 100, 120, and 140 °C. At all annealing temperatures, the PLA-CA composite membranes had a fibrous microstructure, and at their intersection points, the fibers started to interlink. The membrane under the 30 min–80 °C condition had an average fiber diameter of 589 nm and an average pore size of 2.258 μm. At the annealing temperature of 100 °C, the fibers had an average diameter of 649 nm and an average pore size of 1.224 μm. As the annealing temperature increased from 80 to 100 °C, due to PLA-CA composite chain relaxation, the fiber diameter increased. As we further increased the temperature to 120 °C, the average fiber diameter decreased to 528 nm and the pore size was reduced to 1.01 μm. The average fiber diameter decreased due to a change in chain orientation and an increase in crystallinity in the composite microstructures, as validated by the XRD graphs which will be considered later in Figure 12, and it is also discussed in [23]. At the annealing temperature of 140 °C, the average fiber diameter was 578 nm and the average pore size was 0.251 μm. Under the annealing time of 60 min, as shown in Figure 5a–d, and at the annealing temperature of 80 °C, the membrane had a minimum fiber diameter of 463 nm, and the pore size was 1.012 μm; this membrane exhibited the maximum mechanical strength and resistance to hydrostatic pressure.

**Figure 6:**
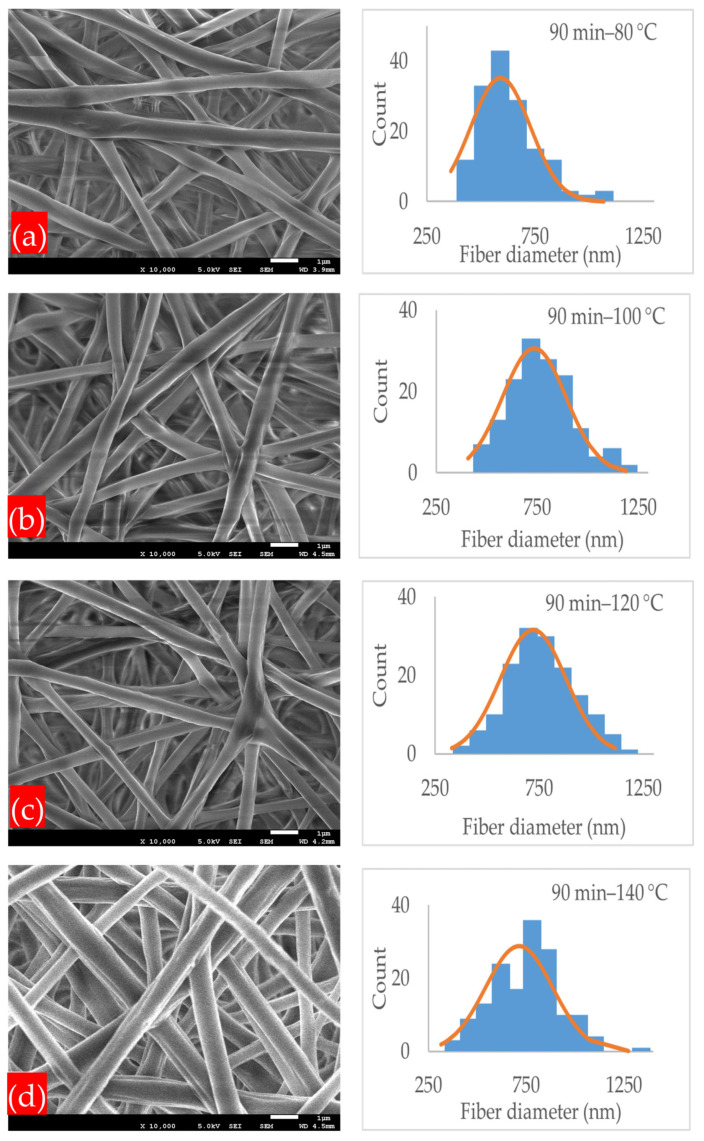
SEM microstructure images and normal distribution of fiber diameter of membranes annealed under following conditions: (**a**) 90 min–80 °C; (**b**) 90 min–100 °C; (**c**) 90 min–120 °C; (**d**) 90 min–140 °C.

By further increasing the annealing temperature to 100, 120, and 140 °C, the average fiber diameter increased to 589, 633, and 687 nm, respectively, and the average pore size was reduced to 0.356, 0.195, and 0.176 μm, respectively. Under the annealing time of 90 min, as shown in Figure 6a–d, and at an annealing temperature of 80 °C, the membrane had an average fiber diameter of 589 nm and a pore size of 0.237 μm, and at 100 °C, the fiber diameter increased to 733 nm.

**Figure 7:**
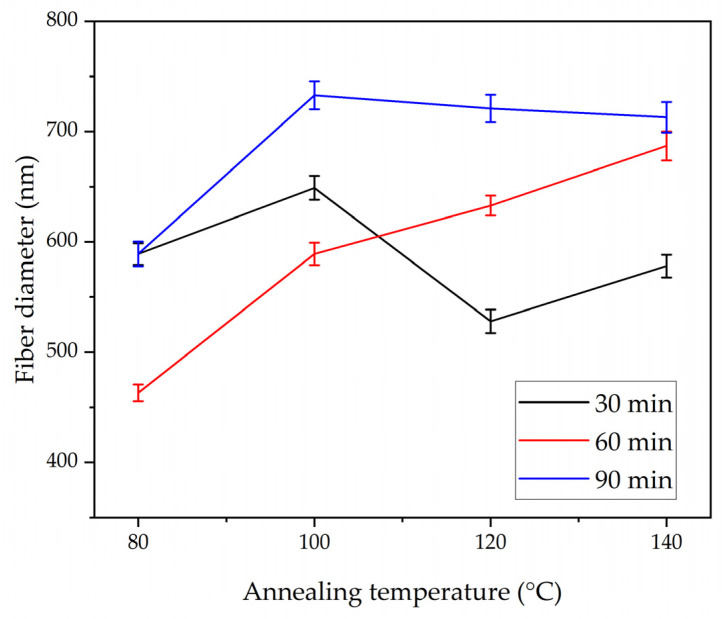
Average fiber diameter of annealed composite membranes under annealing times of 30, 60, and 90 min and annealing temperatures of 80, 100, 120, and 140 °C.

**Figure 8:**
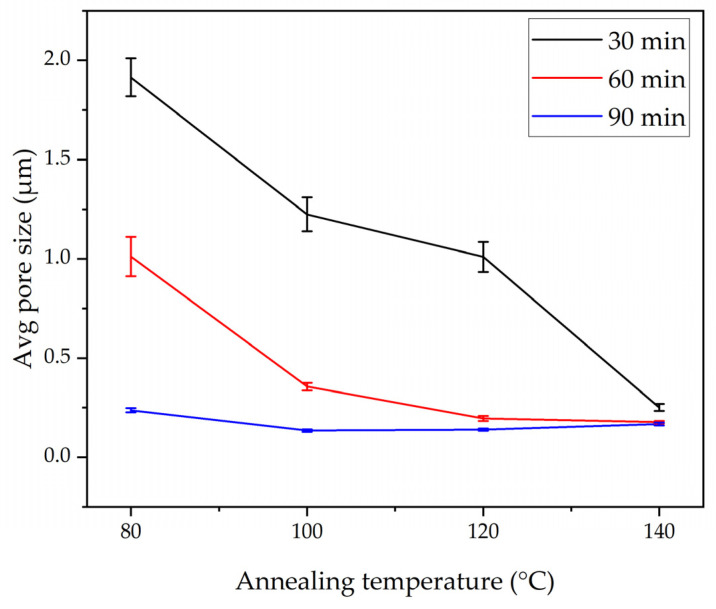
Average pore size of annealed composite membranes under annealing times of 30, 60, and 90 min and annealing temperatures of 80, 100, 120, and 140 °C.

At annealing temperatures of 120 and 140 °C, the mats had an average fiber diameter of 721 and 713 nm, and the average pore size was reduced to 0.1385 and 0.1675 μm, respectively. In general, the annealed membranes had a fibrous network structure. Increasing the annealing time and temperature within the designated annealing conditions did not strongly affect the microstructure, which still remained porous, and no shrinkage or deterioration was observed in the microstructure. A PLA-CA composite electrospun membrane with a fibrous structure and without shrinkage under an annealing time of 90 min and at an annealing temperature of 140 °C was successfully developed. Figure 7 and Figure 8 summarize the effect of annealing on the average fiber diameter and pore size of the composite membranes.

### 3.2. Thermal Properties

**Figure 9:**
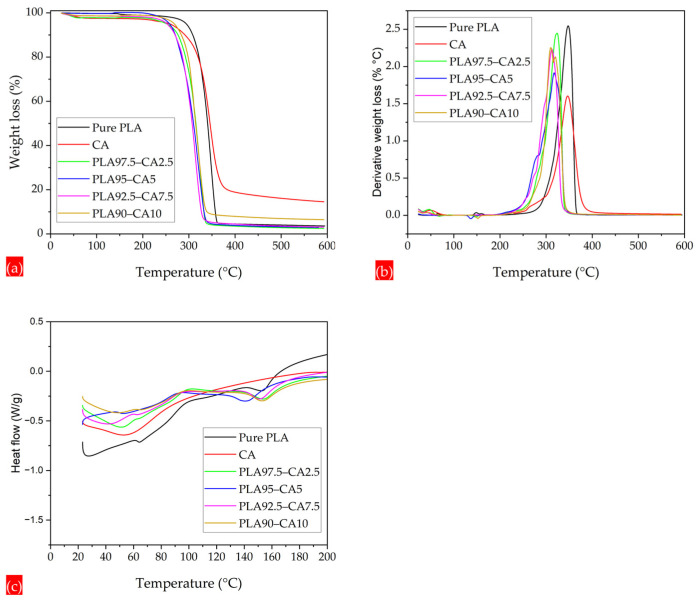
Thermograms of pristine PLA, CA, and PLA-CA composite membranes; (**a**) TGA curves; (**b**) DTG curves; (**c**) DSC curves.

To analyze the thermal stability and transitions of the PLA-CA as-spun and annealed composite membranes, a comprehensive thermal analysis was performed by utilizing thermogravimetric analysis (TGA) and differential scanning calorimetry (DSC). To optimize the polymeric processing system, the characteristics of thermal degradation and the threshold temperature for polymer decomposition are critical [48]. TGA thermograms of weight loss and differential thermogravimetry (DTG) curves were utilized to measure the temperature at different thermal degradation points; the curves showing the maximum temperature for peak weight loss are shown in Figure 9a,b.

Based on the TGA graphs of as-spun PLA-CA and annealed composite specimens, the region for major weight loss of polymer was analyzed between 230 and 350 °C. Due to the hydrophobic nature of the composite membranes, no dehydration was observed around 100 °C. For all the thermograms, the onset temperature (T_on-set_), temperatures at 5% (T_5% WL_), and 50% (T_50% WL_) weight loss were analyzed. The onset temperature (T_on-set_) was measured at the intersection point of the line extending from the pre-degradation portion of the curve and the line at the steepest portion of the mass degradation curve. T_d-max_ represents the peak maximum temperature on the DTG curve. The pristine PLA electrospun membrane had T_on-set_, T_5% WL_, T_50% WL_, and T_d-max_ temperatures of 322.24, 294.45, 340.5, and 347.53 °C. As for the CA solution blended with PLA solution, the composite PLA 97.5–CA 2.5 specimen had T_on-set_, T_5% WL_, T_50% WL_, and T_d-max_ temperatures of 298.2, 254.16, 315.46, and 323.36 °C, respectively. The thermal stability of the composite membranes was reduced compared to pristine PLA. It is reported that the thermal stability of PLA-based composites reduces with the addition of pure cellulose [49]. The PLA 95–CA 5 composite demonstrated the maximum thermal stability among the series of PLA-CA composites. It had a T_on-set_, T_5% WL_, T_50% WL_, and T_d-max_ of 298.9, 271.35, 322.27, and 329.65 °C, respectively. The PLA 92.5–CA 7.5 composition had a lower thermal stability, whereas the PLA 90–CA 10 composite had a relatively high thermal stability. As the concentration of CA in the composite increased, the TGA and DTGA shifted to lower temperatures, as shown in Figure 9a,b. The thermal characteristics of the as-spun composite membranes are summarized in Table 1.

DSC curves were used to calculate the glass transition and melting temperatures of all the composite membrane specimens. Pristine PLA had a glass transition (T_g_) and melting temperature (T_m_) of 61.44 and 146.49 °C, respectively, consistent with what is reported in the literature [50]. The PLA 97.5–CA 2.5 composite had T_g_ and T_m_ temperatures of 60.8 °C and 143 °C, respectively. As the concentration of CA increased in the composite, the glass transition and melting temperatures kept increasing. PLA 90–CA 10 had T_g_ and T_m_ temperatures of 63.24 °C and 147.81 °C, respectively. Figure 9c shows the curves for all the different composite membranes, with T_g_ and T_m_ marked by local changes in the curvature of the curves.

**Figure 10:**
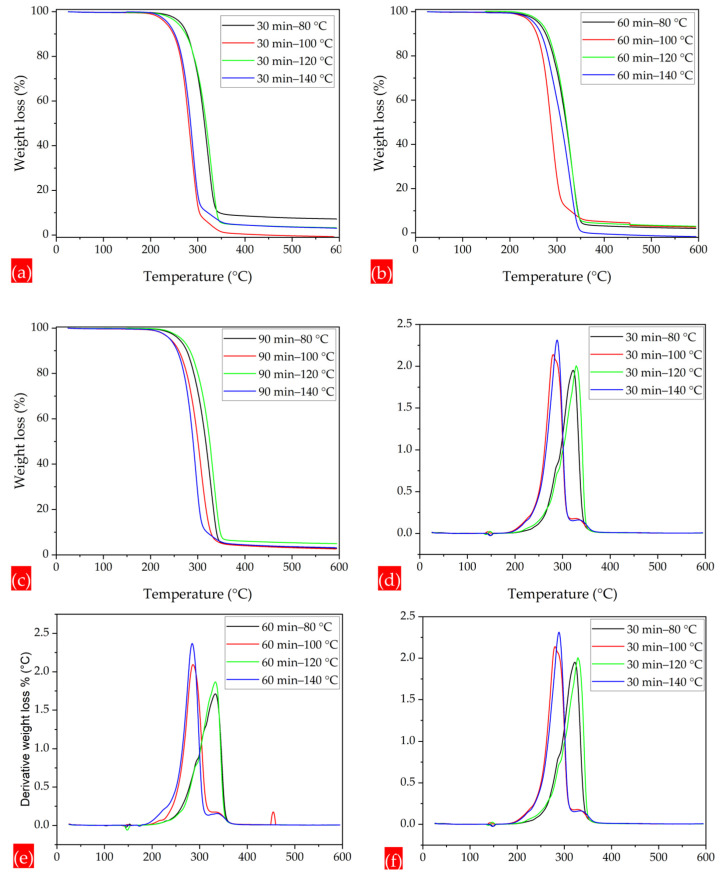
TGA and DTG thermograms of membranes under different annealing times and temperatures, (**a**) TGA thermogram under 30 min and 80 to 140 °C. (**b**) TGA thermogram under 60 min and 80 to 140 °C. (**c**) TGA thermogram under 90 min and 80 to 140 °C. (**d**) DTG thermogram under 30 min and 80 to 140 °C. (**e**) DTG thermogram under 60 min and 80 to 140 °C. (**f**) DTG thermogram under 90 min and 80 to 140 °C.

In the annealing process, as either annealing time or annealing temperature is increased, the polymer chains tend to absorb more energy and start to vibrate vigorously. This enhanced vibrational motion likely led to the relaxation of any internal stresses or constraints that may have been generated in the fiber chains during the electrospinning process. Polymer chains undergo recrystallization and adopt more energetically favorable configurations. With annealing for 30 min, the thermal properties at annealing temperatures of 80 °C and 120 °C are relatively high, as compared to the annealing temperatures of 100 °C and 140 °C, which have thermograms at lower temperatures, as demonstrated in the TGA and DTG thermograms. Similarly, the thermal characteristics under annealing times of 60 and 90 min and at different annealing temperatures, such as 80, 100, 120, and 140 °C, are shown in Figure 10a–f. The thermal analysis reveals the effect of blending CA with PLA on the thermal stability and transitions of PLA-CA composite membranes. The thermal characteristics of the annealed composite membranes are summarized in Table 2.

**Figure 11:**
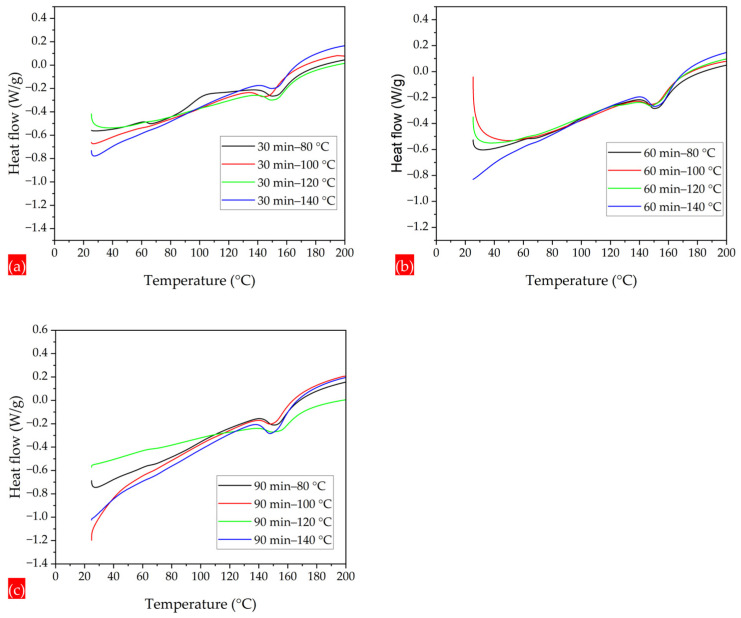
DSC thermograms demonstrating glass transition of annealed membranes. (**a**) DSC thermograms under 30 min and 80 to 140 °C. (**b**) DSC thermograms under 60 min and 80 to 140 °C. (**c**) DSC thermograms under 90 min and 80 to 140 °C.

The DSC thermograms demonstrate the effects of the annealing parameters on the glass transition and melting temperatures. For all annealing times, i.e., 30, 60, and 90 min, and at the annealing temperature of 80 °C, there is a clear visible curve for the glass transition temperature at 63.05, 63.39, and 63.68 °C, respectively. By increasing the annealing temperatures to 100, 120, and 140 °C, there is a reduction in endothermic peaks, and the curves start to straighten. In the DSC thermograms shown in Figure 11a–c, there is a visible reduction in endothermic peaks, due to an increment in crystallinity. The straightening of the DSC curves of the PLA-CA composite samples indicates a reduction in the rate of thermal decomposition. The more relaxed and ordered microstructure may be less susceptible to thermal degradation. Overall, there is an increasing trend in the glass transition temperature due to the annealing process, resulting in an increase in rigidity and relatively more ordered polymer structures. It is important to note that in calculations of such curves, inaccuracies are frequently associated with computations, as the numerical derivative relies on the smoothness of the experimental data [48].

### 3.3. X-Ray Diffraction (XRD) Analysis

**Figure 12:**
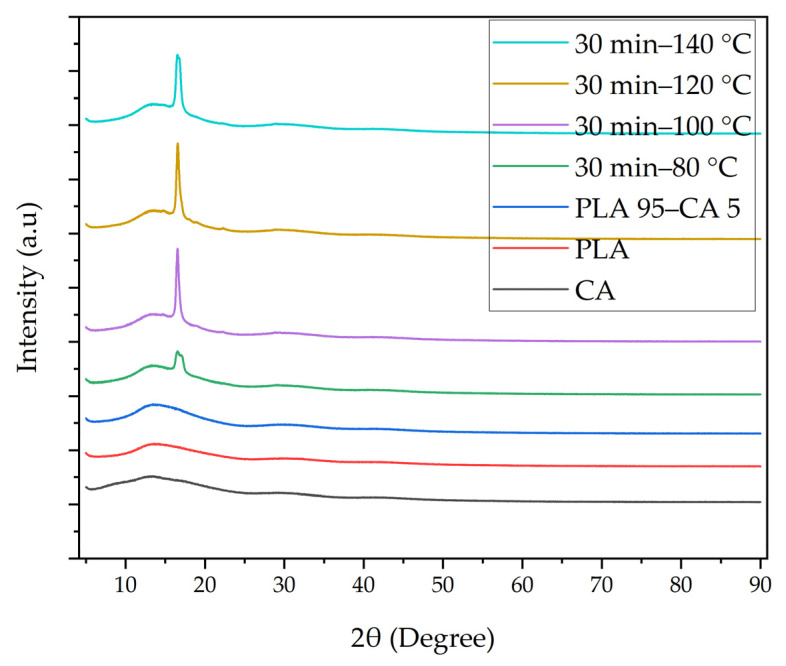
XRD graph of pristine PLA, CA, PLA 95–CA 5, and annealed composite membranes under 30 min and temperatures of 80 to 140 °C.

The crystallinity of the pristine and composite membranes was analyzed with X-ray diffraction (XRD) curves, as shown in Figure 12. The XRD curves of PLA and CA have broad peaks at Bragg’s angles 14° and 13.2°, respectively. This demonstrates the amorphous behavior of the PLA and CA pristine membranes. The PLA-CA composite membrane also shows a broadened peak at a Bragg’s angle of 13.5°, but relatively high intensity as compared to the pristine PLA and CA membranes. For the membrane annealed under 30 min–80 °C, the peak shifts to high intensity, demonstrating a diffraction peak at a Bragg’s angle of 16.6°, corresponding to (110)/(200) crystal planes [23], indicating the crystalline behavior of the composite membrane. For annealing under 30 min–100 °C, the curve has a sharp diffraction peak at a Bragg’s angle of 16.6°. Under a further increase of the annealing temperature to 120 °C, the composite membrane has a sharp peak at a Bragg’s angle of 16.6°, and it has a higher intensity as compared to annealing at 80 °C and 100 °C. With a further increase in the annealing temperature of the composite membrane to 140° C, the XRD curve demonstrates a less sharp peak at 16.6° and a relatively low intensity, as compared to annealing at 120 °C. As the annealing temperature increases, the crystallinity of the membrane increases up to 120 °C; a further increase in the temperature beyond this causes a reduction in the crystallinity of the composite membranes.

### 3.4. Mechanical Properties

**Figure 13:**
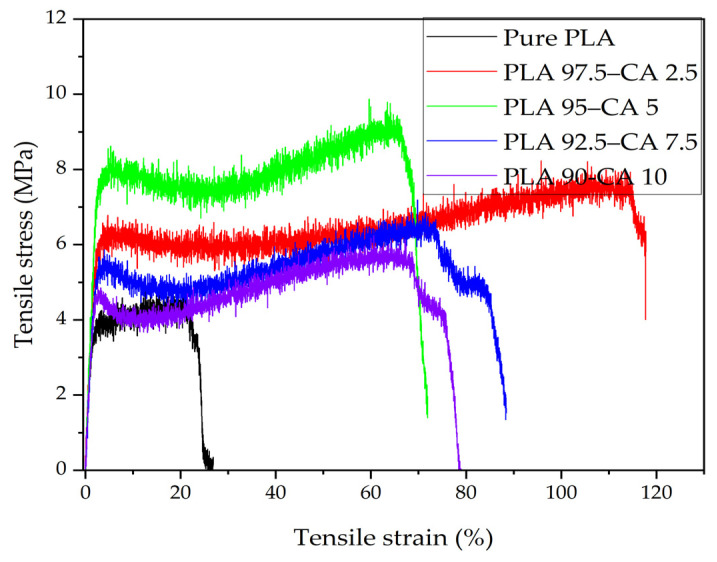
Tensile stress–strain curves for pristine PLA and PLA-CA composites at different concentrations of CA, varying from 2.5 to 10% CA.

The mechanical properties of electrospun nanofibers may be characterized either by consideration of a single electrospun nanofiber or of the membrane specimen. However, it is quite a challenging task to analyze the mechanical characteristics of single fiber [51,52,53]. Most researchers analyze the properties of electrospun nanofiber membranes by utilizing a universal testing machine, which has the capabilities to test most mechanical properties [54,55]. Tensile characteristics determine how a material responds to axially applied loading. Figure 13 demonstrates the stress–strain curves of the pristine PLA and PLA-CA composite membranes.

The tensile strength of the pristine PLA electrospun membrane is 4.27 MPa, its Young’s modulus is 201.4 MPa, and its elongation at break is 26.8%, which demonstrates the brittle behavior of PLA. These results are consistent with those reported in the literature [55]. Generally, the fiber diameter has a strong effect on the tensile strength and Young’s modulus [56]; as the fiber diameter decreases, the mechanical properties are enhanced. The PLA 97.5–CA 2.5 composite exhibits a tensile strength of 8.07 MPa, a Young’s modulus of 252.5 MPa, and a maximum elongation of 117.8% at break point. When the CA concentration is increased to 5%, the PLA 95–CA 5 composite membrane exhibits superior tensile behavior, with a tensile strength of 9.3 MPa and a Young’s modulus of 291.1 MPa, while the elongation at break is reduced to 72.9%, which can be explained by it having superior nano characteristics and a minimum average fiber diameter.

**Figure 14:**
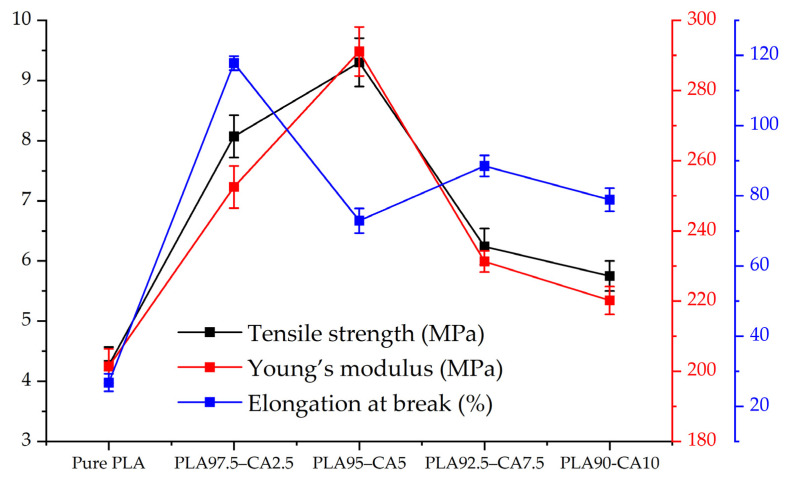
Tensile strength, Young’s modulus, and elongations at break of pristine PLA and PLA-CA composites at different concentrations of CA, varying from 2.5 to 10% CA.

With a further increase in the concentration of CA, due to the relative increase in fiber diameter, there is a reduction in the tensile strength and Young’s modulus. The PLA 92.5–CA 7.5 composite has a tensile strength of 6.24 MPa, a Young’s modulus of 231 MPa, and an elongation at break of 88.5%, while for the PLA 90–CA 10 system, the tensile strength is 5.75 MPa, the modulus of elasticity is 220 MPa, and the elongation at break is 78.9%. Figure 14 summarizes the tensile strength, Young’s modulus, and elongation at break of the pristine PLA and PLA-CA composite mats at different concentrations.

The PLA 95–CA 5 as-spun composite membrane demonstrated superior tensile strength among all the composites. It was selected as the optimal composite membrane, and all the annealing treatments were performed on the PLA 95–CA 5 composite membrane. The annealing process enhances the crystallinity, molecular rearrangements, and physical interactions between the fibers of electrospun composite membranes, which causes an increment in the tensile strength and ductility of composite membranes. However, these characteristics increase up to a specific temperature, but a further increase in the annealing temperature causes an increase in the rigidity of the fibers and a reduction in crystallinity, which is demonstrated by DSC and XRD analysis, and was discussed earlier. Under an annealing time of 30 min and an annealing temperature of 80 °C, the composite mat has a tensile strength of 12.1 MPa, a Young’s modulus of 368 MPa, and an elongation at break of 96.6%. Increasing the annealing temperature to 100 °C, the tensile stress–strain curve shows that the tensile strength, Young’s modulus, and elongation at break slightly decrease to 11.5 MPa, 343 MPa, and 74%, respectively.

**Figure 15:**
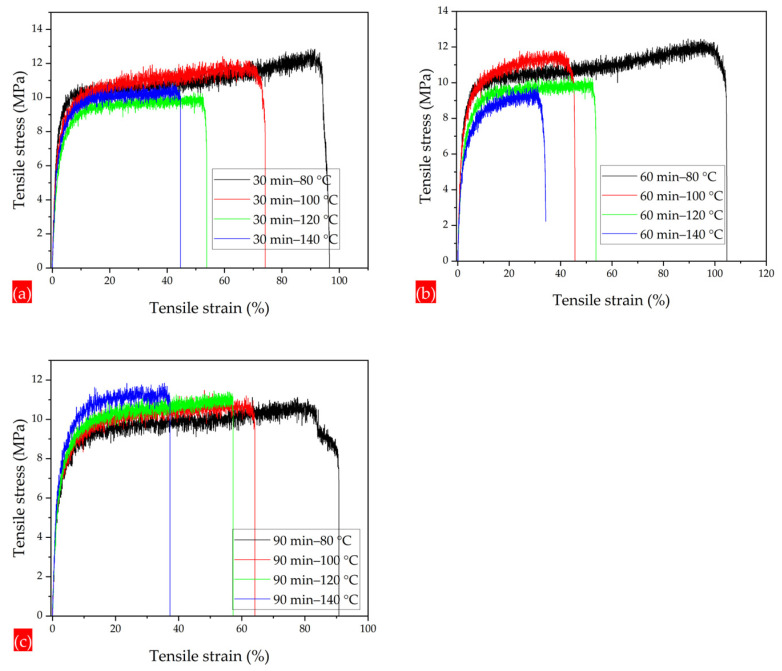
Stress–strain curves of membranes (**a**) annealed under 30 min and 80 to 140 °C, (**b**) annealed under 60 min and 80 to 140 °C, and (**c**) annealed under 90 min and 80 to 140 °C.

**Figure 16:**
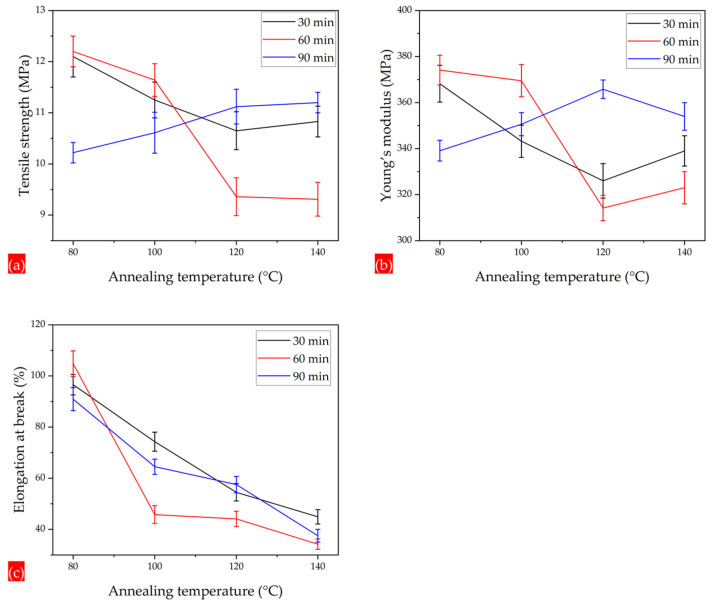
(**a**) Tensile strength of membranes under annealing times of 30, 60, and 90 min and annealing temperatures of 80, 100, 120, and 140 °C. (**b**) Young’s modulus of membranes annealed under times of 30 to 90 min and temperatures of 80 to 140 °C. (**c**) Elongation at break of membranes annealed under times of 30 to 90 min and temperatures of 80 to 140 °C.

With a further increase in the annealing temperature to 120 °C, the mat has a tensile strength of 9.8 MPa, a Young’s modulus of 326 MPa, and an elongation at break of 54%. At the highest annealing temperature of 140 °C, there is a increment in the tensile strength to 10 MPa and in the Young’s modulus to 339 MPa. However, there is a significant reduction in elongation at the break point, which is 45%. Initially, by increasing the annealing temperature, the crystallinity increases, but a further increase in temperature tends to enhance the rigidity of electrospun fibers, which may increase the tensile strength and Young’s modulus, and cause a prominent reduction in elongation at the break point, as shown in Figure 15a. Under the annealing time of 60 min and annealing temperature of 80 °C. the membrane has superior mechanical properties, and the curve shows a maximum tensile strength of 12.2 MPa, a Young’s modulus of 374 MPa, and an elongation at break of 104.8%. At the temperature of 100 °C, the membrane has a tensile strength of 11.8 MPa, a Young’s modulus of 369 MPa, and an elongation at break of 45%. By further increasing the annealing temperatures to 120 °C and 140 °C, it has tensile strength of 9.4 MPa and 9.46 MPa, and the elongation at break is reduced from 44 to 34%, respectively, as shown in Figure 15b. Under the annealing time of 90 min and temperature of 80 °C, the mat has a tensile strength, Young’s modulus, and elongation at break of 10.2 MPa, 339 MPa, and 91%, respectively. As we further increase the annealing temperature to 100, 120, and 140 °C, the tensile strength increases, and the elongation at break is prominently reduced, as shown in Figure 15c. Figure 16a–c summarizes the tensile strength, Young’s modulus, and elongation at break of the composite membranes annealed under temperatures of 80, 100, 120, and 140 °C and times of 30, 60, and 90 min.

### 3.5. Hydrophobicity

The hydrophobicity of the as-spun and annealed composite membranes was analyzed by measurement of the water contact angle (WCA). In WCA measurements, the dependency of the contact angle on the deionized water drop size is neglected. It is reported that when the drops’ base diameter is between 1 and 7 mm, the contact angle is not significantly affected by the drop size [52]. The contact angle is measured from a digital video image of drops on the membrane with the help of image processing software, which allows for the calculation of the WCA from the circle fitting of the water droplets utilizing the sessile drop method.

**Figure 17:**
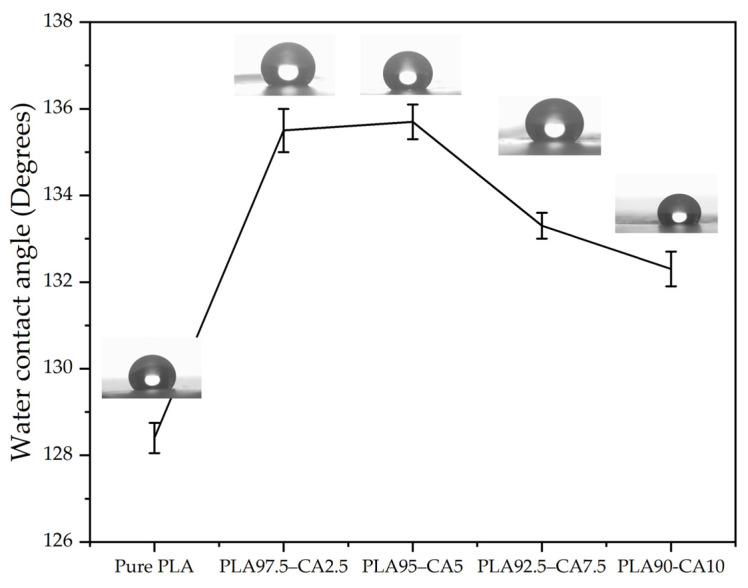
Water contact angle of as-spun pristine PLA and PLA-CA composites at different concentrations of CA, varying from 2.5 to 10% CA.

To minimize error, multiple readings were taken at different locations on the membrane. Furthermore, each measurement lasted over two minutes, to avoid the effects of variations in measurement of contact angle over time due to liquid evaporation. The water contact angle of the as-spun pristine PLA membrane was 128.1°. The PLA 97.25–CA 2.5 and PLA 95–CA 5 composite membranes had WCAs of 135.5° and 135.7°, respectively. A further increase in the concentration of CA decreased the WCA, with values for the PLA 92.5–CA 7.5 and PLA 90–CA 10 systems being 133 and 131.8°, respectively. Electrospun cellulose acetate is hydrophilic in nature; as the concentration of CA increases, the hydrophobicity of the composite membrane decreases, as shown in Figure 17.

**Figure 18:**
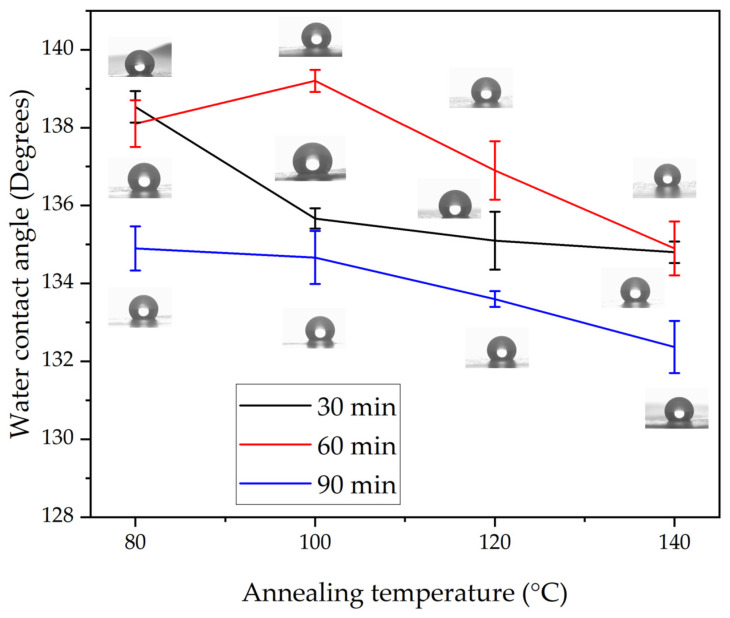
Water contact angle of membranes annealed under times of 30 to 90 min and temperatures of 80 to 140 °C.

Among the composite members, the PLA 95–CA 5 specimen demonstrated superior morphological, thermal, and mechanical properties, and also showed higher hydrophobicity. Further, the effect of annealing on the hydrophobicity of PLA 95–CA 5 was analyzed. Figure 18 shows the water contact angles under different annealing parameters. Under an annealing time of 30 min and a temperature of 80 °C, the membrane has WCA of 138.5°, and as the annealing temperatures keep increasing, the WCA decreases. At the annealing temperatures of 100, 120, and 140 °C, the composite membranes have WCAs of 135.7, 135.1, and 134.8°, respectively. Under the annealing time of 60 min and temperature of 80 °C, the composite has a WCA of 138.1°, and at a temperature of 100 °C, the composite membrane demonstrates the highest WCA of 139.2°. With a further increase in the annealing temperatures to 120 °C and 140 °C, the WCA decreases to 136.9° and 134.9°, respectively. Under the annealing time of 90 min and annealing temperature of 80 °C, the WCA is 134.9°. By increasing the annealing temperatures to 100, 120, and 140 °C, the WCA decreases to 134.7, 133.6 and 132.4°, respectively. Generally, by increasing the annealing parameter of either time or temperature, the WCA decreases. The macroscopic wetting characteristics of solid surfaces are highly dependent on their surface morphology and surface energy [57]. The changes in the water contact angle can be attributed to changes in the microstructure of the electrospun membranes induced by the annealing process, including the molecular rearrangement, surface roughness, and porosity of the membranes.

### 3.6. Analysis of Resistance to Hydrostatic Pressure and Breathability 

**Figure 19:**
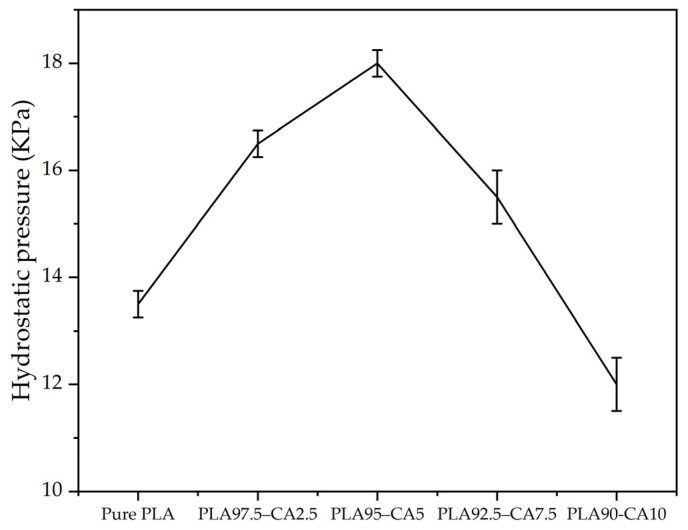
Resistance to hydrostatic pressure of as-spun pristine PLA and PLA-CA composites at different concentrations of CA, varying from 2.5 to 10% CA.

The strength and waterproofness of the as-spun and annealed PLA-CA composite membranes were also validated by tests of resistance to hydrostatic pressure. A manual setup is developed for testing the resistance to hydrostatic pressure. Air suction pressure was applied at the bottom end of the membrane, and at the top end, water was poured on glass. The pressure on the membrane was developed by gradually increasing the suction pressure. The resistance to hydrostatic pressure was noted at the point when water droplets started coming down from the bottom of the membrane. The pure PLA mat resisted a hydrostatic pressure of up to 13.5 KPa. The PLA 97.5–CA 2.5 and PLA 95–CA 5 specimens withstood a hydrostatic pressure of 16.5 and 18 KPa. With a further increase in the concentration of CA, the hydrostatic pressure decreased. The PLA 92.5–CA 7.5 and PLA 90–CA 10 specimens withstood hydrostatic pressures of 15.5 and 10 KPa, respectively, as shown in Figure 19.

**Figure 20:**
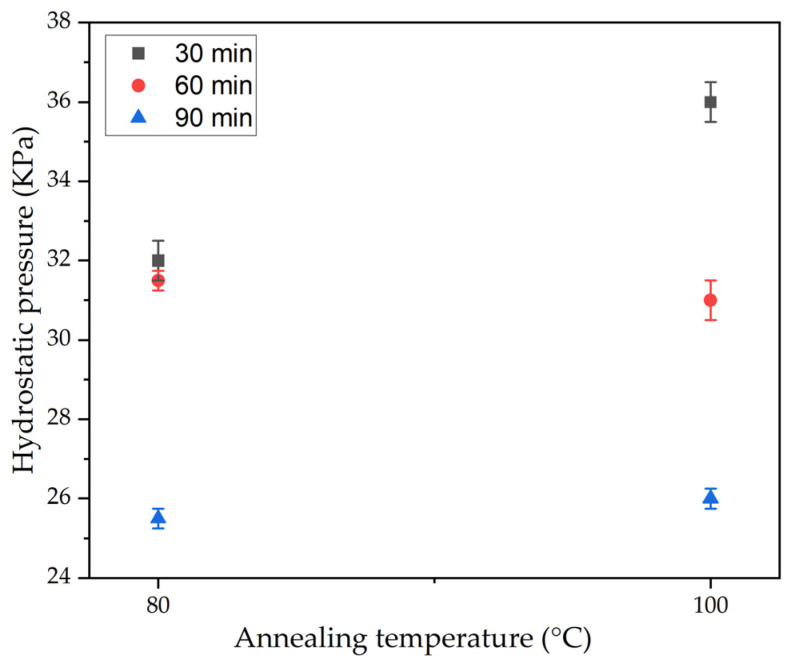
Resistance to hydrostatic pressure of membranes annealed under times of 30, 60, and 90 min and temperatures of 80 °C and 100 °C.

In resistance to hydrostatic pressure testing, the PLA 95–CA 5 composite membrane maintained its superiority over the other composite membranes by demonstrating higher resistance to hydrostatic pressure. Annealing of the PLA 95–CA 5 composite membrane further enhanced the strength and waterproofness of the composite membrane, which is also verified by the resistance to hydrostatic pressure results. Annealing at temperatures of 80 °C and 100 °C provided better mechanical properties, so we performed the hydrostatic testing only at temperatures of 80 °C and 100 °C. The membrane annealed under 30 min–80 °C showed a resistance to hydrostatic pressure of 32 KPa, while the membrane annealed at 100 °C had a resistance to hydrostatic pressure of 36 KPa. The mats under 60 min of annealing time and annealing temperatures of 80 °C and 100 °C showed a resistance to hydrostatic pressure of 31.5 and 31 KPa, respectively. These improvements in hydrostatic pressure are attributed to increases in crystalline structure, improvements in morphology, and enhanced mechanical properties. As we further increased the annealing time to 90 min, the resistance to hydrostatic pressure of the mats decreased; they withstood 25.5 and 26 KPa of hydrostatic pressure at 80 °C and 100 °C, respectively. The hydrostatic test results of the annealed samples are shown in Figure 20.

**Figure 21:**
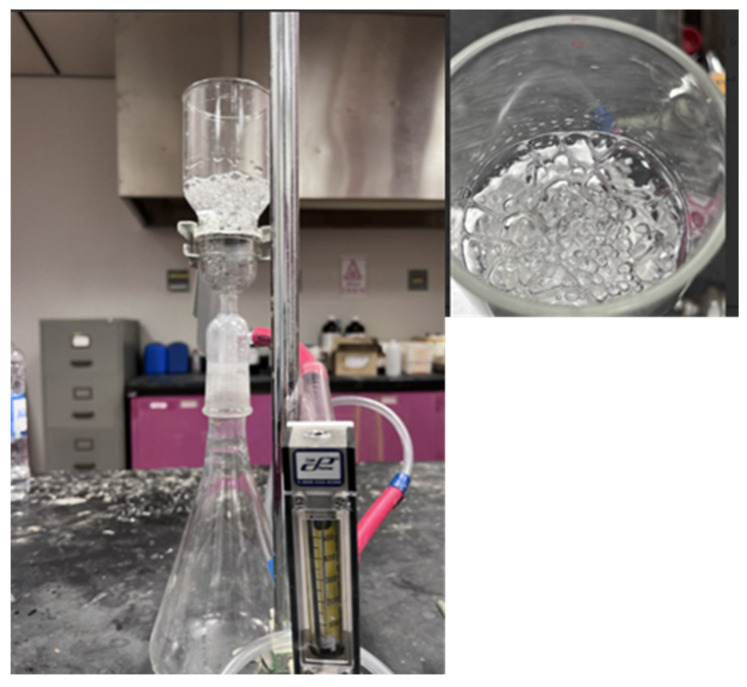
Breathability of as-spun and annealed composite membranes.

The breathability of the as-spun and annealed composite membranes was analyzed by passing compressed air through the membrane while the membrane had water on its top surface, as shown in Figure 21. All the as-spun and annealed composite membranes showed good breathability, as bubbles were clearly visible, indicating the passing of air through the membrane, while there were no water droplets going down through the membranes.

### 3.7. FT-IR Spectroscopy

**Figure 22:**
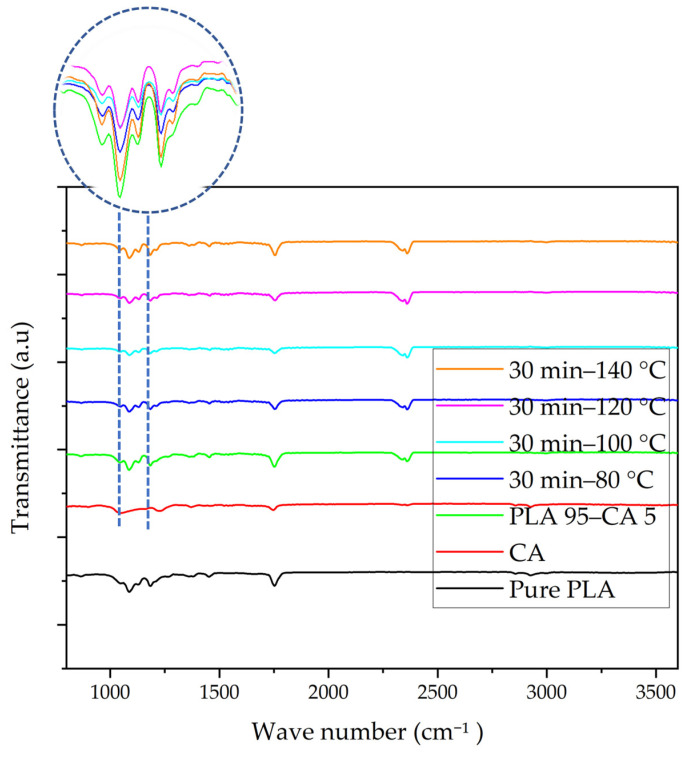
FT-IR spectra of pristine PLA, CA, PLA 95–CA 5 composite, and membranes annealed under 30 min and temperatures of 80 to 140 °C.

FT-IR analysis was used to analyze the functional groups attached to the pristine PLA, CA, as-spun, and annealed composite membranes in the range of 500–4000 cm^−1^, as shown in Figure 22. The FT-IR curve of pristine PLA shows the vibrational spectrum of the electrospun PLA membrane. In the graph, the peak at 1087 cm^−1^ represents C-O stretching. The bands at 1755 and 1184 cm^−1^ show the stretching vibration of the carbonyl (C=O) and C-O, indicating the presence of the ester group which is the main building block of PLA. The peak at 2930 cm^−1^ represents the C-H stretching vibrations in the methylene (-CH₂) groups, and confirms the presence of aliphatic hydrocarbon chains in PLA. The FT-IR spectrum is consistent with what is reported in [49]. In the CA graph, the band at 1047 cm^−1^ is associated with stretching of C-O-C in cellulose acetate, and the bands at 1747 and 1236 cm^−1^ represent the carbonyl (C=O) and C-O stretching vibrations in the ester groups. The peak at 2927 cm^−1^ represents C-H stretching in the methylene (-CH₂) groups; the spectrum is consistent with [58]. The FT-IR spectrum of the PLA 95–CA 5 composite demonstrates bands at 1751 cm^−1^ and 1184 cm^−1^, representing the carbonyl (C=O) and C-O stretching vibration in the ester groups. The bands at 1087 cm^−1^ and 2996 cm^−1^, related to C-O and C-H stretching vibrations, represent both PLA and CA in the polymer. The peak at 2364 cm^−1^ in the spectrum indicates the presence of carbon dioxide in the atmosphere. The broader peaks appearing between 3400 and 3700 cm^−1^ are characteristic of hydroxyl (O-H) groups, representing hydrogen bonding or interactions between PLA and CA.

The spectra of the annealed samples show peaks at 1087 cm^−1^ and 1184 cm^−1^, representing stretching of carbon-oxygen-carbon (C-O-C) bonds and stretching of carbon-oxygen (C-O) bonds. The peak at 1754 cm^−1^ is characteristic of the carbonyl (C=O) group. The peak at 2996 cm^−1^ represents the stretching of carbon–hydrogen (C-H) bonds in polymer composites. The broader small peaks represent hydrogen bonding between PLA and CA. After the annealing process, there was a slight shift in peak position, and an increase in the intensity of peaks; this represents changes in the molecular structure due to an increase in crystallinity. None of the annealing spectra showed any major shift in peaks or the appearance of new functional groups after the annealing treatment, which indicates that there was no decomposition of polymer components due to annealing.

## 4. Conclusions

Environmentally friendly and biodegradable biopolymer membranes were successfully fabricated via the electrospinning technique and modified through solution blending and annealing techniques. The developed composite nanofibrous membranes were characterized by FE-SEM, XRD, FR-IT, TGA, DSC, tensile testing, water contact angle analysis, breathability analysis, and testing of resistance to hydrostatic pressure. In the first phase, during the pre-electrospinning process, cellulose acetate solution was blended with polylactic acid solution at different concentrations. The pristine PLA membrane had an average fiber diameter of 746.3 nm and a water contact angle of 128.1°. CA solution was blended into PLA solution at different concentrations, such as 2.5, 5, 7.5, and 10% (*v/v*) concentrations. The concentration of CA in the solution influenced the fiber morphology; by increasing the CA concentration from 0 to 5%, the average fiber diameter decreased, and the PLA 95–CA 5 composite had the lowest fiber diameter of 432.1 nm. Due to its superior nano characteristics, the PLA 95–CA 5 composite showed the maximum tensile strength of 9.3 MPa, a Young’s modulus of 291 MPa, and an elongation at break of 72.9%. It showed a water contact angle of 135.7° and a resistance to hydrostatic pressure of 18 KPa. By further increasing the concentration of CA in the blend solution, there was an increase in the average fiber diameter, and a reduction in the tensile strength, Young’s modulus, elongation at break, and thermal stability. The water contact angle and hydrostatic pressure were also reduced due to an increase in the concentration of CA. In the blending phase, PLA 95–CA 5 was selected as the optimal sample, based on its morphology, mechanical strength, thermal stability, hydrophobicity, and hydrostatic pressure.

In the second phase, annealing was performed on the optimal PLA 95–CA 5 composite membrane. Annealing parameters such as annealing time and temperature were selected to achieve the best compromise between morphological, thermal, mechanical, and hydrophobic characteristics, based on DSC graphs and FE-SEM microstructural images. In the annealing process, the annealing temperatures were varied from 80 °C to 140 °C, and the annealing times were varied from 30 min to 90 min. These annealing parameters influenced the morphological, mechanical, thermal, and hydrophobic properties of the composite membranes. During the annealing process, the fibers rearranged themselves in favorable directions, increasing the crystallinity and rigidity of the electrospun fibers. The most lucrative result of this research is that we obtained thermally stable composite membranes with enhanced mechanical properties and fibrous microstructures, without any deterioration under an annealing temperature of 140 °C and annealing time of 90 min. The annealing process improved the mechanical properties, thermal stability, and resistance to hydrostatic pressure to a certain extent; beyond this limit, due to an increment in the rigidity of the nanofibers, these properties started to reduce. The composite membranes were shown to have a fibrous microstructure under all annealing parameters. The composite membranes annealed under 80 °C and 120 °C showed higher thermal stability as compared to membranes annealed under 100 °C and 140 °C. An increase in annealing temperature enhanced the crystallinity of the composite membranes, which showed a sharp crystalline peak at a Bragg’s angle of 16.6°. The annealed composite membranes showed a maximum tensile strength of 12.2 MPa, a Young’s modulus of 374 MPa, and an elongation at break of 104.8%. They showed enhanced hydrophobicity, with a water contact angle of 139.2°, an improvement in resistance to hydrostatic pressure of 36 KPa, and good breathability characteristics. FT-IR analysis revealed the functional groups attached to the as-spun and annealed composite membranes.

## Figures and Tables

**Table 1:** Thermal properties of PLA-CA composite membranes.

Composite Membrane	T_on-set_ (°C)	T_5% weight loss_ (°C)	T_50% weight loss_ (°C)	T_d-max_ (°C)	T_g_ (°C)	T_m_ (°C)
PLA	322.24	294.45	340.55	347.53	61.44	146.49
CA	321.11	261.05	348.8	349.86	213.84	
PLA 97.5–CA 2.5	298.19	254.16	315.46	323.36	60.8	143.02
PLA 95–CA 5	298.91	271.35	322.27	329.65	61.36	144.24
PLA 92.5–CA 7.5	288.13	255.03	308.25	313.54	62.44	145.57
PLA 90–CA 10	293.55	270.2	314.54	310.2	63.24	147.81

**Table 2:** Thermal properties of annealed PLA-CA composite membranes.

Composite Membrane	T_on-set_ (°C)	T_5% weight loss_ (°C)	T_50% weight loss_ (°C)	T_d-max_ (°C)	T_g_ (°C)	T_m_ (°C)
30 min–80 °C	290.6	263.6	314.2	322.3	63.1	144.3
30 min–100 °C	263.4	237.7	286.1	285.5	64.5	144.9
30 min–120 °C	295.5	257.3	318.4	329.5	65.6	144.3
30 min–140 °C	264	236.1	283.8	287.4	64.1	143.5
60 min–80 °C	291.3	260.7	318.8	332.9	63.4	144.5
60 min–100 °C	264.5	245.6	287.6	286.5	64.8	143.2
60 min–120 °C	296.3	265.7	320.1	321.6	64.5	143.5
60 min–140 °C	279.1	250.3	307.4	326.6	63.3	143.5
90 min–80 °C	293.4	260.9	316.8	328.8	63.7	144.2
90 min–100 °C	276.3	244.1	301.7	306.8	64.8	144.5
90 min–120 °C	302.7	266.9	325.1	320.7	65.5	144.3
90 min–140 °C	270.4	242.2	290.1	293.9	64.3	142.8

## Data Availability

The original contributions presented in this study are included in the article. Further inquiries can be directed to the corresponding author.
